# Learning from the Dirt: Initiating university food gardens as a cross-disciplinary tertiary teaching tool

**DOI:** 10.1007/s42322-022-00100-6

**Published:** 2022-05-13

**Authors:** Cathy Sherry

**Affiliations:** UNSW Law and Justice, Scientia Education Academy Fellow, UNSW Sydney, Sydney, Australia

**Keywords:** Campus garden, Environmental education, Urban agriculture, Food systems, Australia, United States

## Abstract

Food gardens are an underdeveloped resource for teaching and research in Australian universities. While some campuses have food or botanical gardens, outside the biological or physical sciences food growing is not routinely incorporated into mainstream curricula. This article investigates why and how we might change this. It examines universities’ traditional reliance on classroom-based, non-experiential learning, which preferences reading and writing over doing, particularly doing anything outdoors. Questions are raised about the implications of this approach for environmental education and graduate environmental literacy. The article then explores the example that United States campus food gardens provide other universities, as well as the process of creating food gardens for teaching and research at a high-density campus in Sydney, Australia. The article ends by postulating lessons students and staff might learn about food systems, sustainability and green cities, when food growing is incorporated into teaching and research. The article aims to inspire academic staff, particularly outside the biological sciences, to instigate campus food gardens to increase graduate environmental literacy.

**Supplementary Information:**

The online version contains supplementary material available at 10.1007/s42322-022-00100-6.

## Introduction

While a common phenomenon on North American university campuses, campus food gardens are rarer in Australia. Gardens can take on a number of forms, as volunteer community gardens for the university or wider community, (Anderson et al., 2018; Marsh et al., 2020, Murkami, 2016), as service-learning gardens (Aftandilian & Dart, 2013), as showcases for university sustainability or as sites for formal teaching and research (Duram & Klein, 2015; Duram & Williams, 2015; Eugenio-Gozalbo et al., 2021**).** While some Australian campuses have food or botanical gardens (Anderson et al., 2018; Marsh et al., 2020), food gardens remain an underdeveloped resource in Australian universities and food growing is not routinely incorporated into mainstream curricula, particularly outside the biological or physical sciences. This article investigate why and how we might change this. Part I examines universities’ traditional reliance on classroom-based, non-experiential learning, which preferences reading and writing over doing, particularly doing anything outdoors. Questions are raised about the implications of this approach for environmental education and graduate environmental literacy. Part II explores the example that food gardens on United States campuses provide other universities, focussing on the role these gardens play in university sustainability programs and environmental curricula. Part III describes a cross-disciplinary project to create food gardens for teaching and research at UNSW, a high-density campus in Sydney, Australia’s largest city. Finally, Part IV postulates lessons students and staff might learn about food systems, sustainability, and green cities, when they get their hands in the dirt.

The article is not an empirical study of the effectiveness of a particular garden(s), rather it is a descriptive and persuasive piece that aims to inspire academics, from all disciplines, particularly those outside biological sciences, to instigate food gardens on their own campuses, in order to increase graduate environmental literacy. Before any empirical work can be conducted on the benefits of university food gardens as teaching tools, food gardens have to be built. As a result, normative research on food gardens – why they might be a good idea, what benefits we postulate they might bring—is a logical precursor to any empirical study.

## Part I: The Importance of Doing: Experiential and outside learning

Despite efforts to include experiential learning in university curricula, the fact remains that significant sections of tertiary teaching and research, typically in arts, law and social sciences, remains firmly focussed on books. Outside of the biological and physical sciences, it is common for whole subjects, even entire degrees, to have no practical component, let alone a component taught outside. There are a range of structural and cultural reasons for this, including research metrics, hiring and promotion policies, as well as deeply held assumptions about what it means to be intellectual or educated.

David Orr, author of the seminal book *Environment in Mind: On Education, Environment, and the Human Prospect* (1994) argues that universities’ failure to value practical knowledge is one of the key reasons we now find ourselves in the midst of an environmental crisis. Orr says that the result of funnelling academically-able students away from vocational courses into theory focussed universities is an entire generation of people who are “technologically illiterate and technologically incompetent” (Orr, 1994, p. 157). A technologically illiterate and incompetent population can readily enjoy the benefits of technology but cannot see its detriments. Orr argues that universities have produced tens of thousands of “highly qualified” graduates who have negligible understanding of the environmental impacts of the innovations and businesses they have been trained to pursue.

Orr notes that the decline in vocational education parallels the decline of people engaged in small-scale farming in the United States. This in turn has led to a growing “ignorance of how ecosystems work and how private consumption and economic growth destroy the environment…However imperfectly, farms served as a reality check on human possibilities in nature that urban societies presently lack” (Orr, 1994, pp. 117–118). This problem is even more accute in Australia, because outside Indigenous communities, Australians have almost never lived en masse off the land. While agriculture was the backbone of the Australian economy, as a result of Australia’s soil and climate, from its inception agriculture typically took the form of large-scale pastoralism, not small-scale farming. Lacking the deep, complex knowledge of Australian land possessed by Indigenous people (Pascoe, 2014), settlers struggled to survive on much rural land in Australia in a way that people did not in the United States. To the frustration of colonial governments, left to their own devices, immigrants often gravitated towards towns (Ford and Roberts, 2013, p. 135). By the mid-twentieth century, half Australia’s population lived in capital cities, and today, we are one of the most urbanised nations on earth, with over 90 per cent of the population living in urban areas (Australian Bureau of Statistics, 2016). Somewhat astoundingly, in 2016 a mere 228,372 people were directly employed in agriculture in Australia, representing only 2.2 per cent of all employed people (Binks et al. 2018). Further, while many urban Australians maintained food-producing backyards throughout the twentieth century (Gaynor, 2006), mirroring the European tradition of allotments, recent decades of urban consolidation have radically reduced housing lot size, with millions of Australians now living in apartments with minimal growing space (Sherry, 2017, p. 10).

The consequence is that most Australian students come to university with little or no experience of living from the land, growing food or other plants. This has flow on effects for environmental literacy because one of the fundamental ways human beings have traditionally gained an appreciation of the precarity of the natural world is through the imperative of growing their own food. Our survival depended on our plants’ survival. Urbanisation has broken this nexus for city-dwellers.

Orr argues that universities can only produce ecologically literate students if students have studied natural systems “roughly in the manner in which we experience them” (Orr, 1994, p. 95). Students need to get outside and be in nature to understand it. He argues that his would remove “the abstractness and second hand learning that corrupts knowledge at its source. Natural objects have a concreate reality that the abstractions of textbooks and lectures do not and cannot have” (1994, p. 95). Orr’s arguments are backed up by research on “plant blindness” (Wandersee & Schussler, 1999), a phenomenon observed in students from primary through to tertiary education which posits that insufficient interaction with the natural environment leads to an inability to notice plants, which in turn leads to a lack of understanding of the importance of plants in the biosphere and for human survival. Research also suggests that people who devote their lives to environmental activism, displaying high levels of environmental literacy, nearly all share common formative experiences, including early interaction with nature (Tanner, 1980), as well as environmentally influential teachers in primary, high school and tertiary education (Chawla, 1998; Lugg, 2007). Most significant memories of environmental education feature “opportunities to take action, rather than passive classroom learning” (Chawla, 1999, p. 21).

Orr is an advocate of campus farms, arguing that if agriculture had been included in the liberal arts curriculum, it would have helped avoid the “deliberate separation of abstract intellect and practical intelligence” (1994, p. 120). He argues that the creation of campus farms would teach students frugality, self-reliance, practical and ecological competence; they would be “interdisciplinary laboratories” which could preserve biodiversity threatened by development; and they could reduce food miles and close waste loops. Campus farms would allow students to participate in the implementation of intelligent, practical solutions that have real impact on sustainability.

There are multiple challenges to creating campus farms (Duram & Klein, 2015; Duram & Williams, 2015; Murkami 2016; Pearson et al., 2005), including a lack of physical space or willingness to prioritise growing space, cost, liability, and above all else, time. Shortened teaching periods, and exponentially increasing quantities of potential reading in a system no longer limited by physical printing, make the decision to cut indoor class time and/or assigned reading difficult. However, if we fully comprehend the importance of *doing*, in addition to reading, the decision becomes easier.

To illustrate this point, consider this example. If someone were to say, ‘I am a very knowledgeable gardener. I have never actually gardened, but I have read many books on the subject,’ people would think that person mildly deluded. However, switch to a university context, and no one would think it was unusual for either students or academics to be considered knowledgeable about urban agriculture or greening cities never having grown a plant. The point should not be overstated; of course, vast amounts can (and must) be learned by reading. By reading, students can learn why Cuba is the poster child for urban agriculture (Gonzalez, 2003), how urban agriculture supports communities in rapidly growing West African cities (Lee-Smith, 2010), whether urban agriculture genuinely alleviates food deserts in the United States, (Siegner et al., 2018). But if they have never grown a vegetable, students may argue that urban growers need “security of tenure,” such as a long-term lease or freehold title. However, while security of tenure is convenient, and will justify greater expenditure on infrastructure, vegetables do not need a perpetual or long-term interest in land; most vegetables are annuals and need to be replanted every 4–9 months. Further, vegetables do not need the entire vertical span of a land lot, which owing to the maxim ‘*cuius est solum eius est usque ad coelum et ad inferos,*’ [whoever owns the soil, it is theirs up to heaven and down to hell] theoretically stretches from the heavens to the centre of the earth below. Vegetables can be grown on slices of the lot, such as roofs and walls. This is because vegetables are shallow rooted, a point of knowledge that is obvious if you have ever pulled up a vegetable but not so obvious if you have not.^1^ Only students who have never grown food argue that cities can be self-sufficient. Had they tried to grow a wind-pollinated grain crop in their backyard or balcony, they would know that it is impossible, and that urban agriculture can only ever *supplement* food supplies, most of which will always come from rural areas.

The next section of the article draws on informal visits to multiple campuses in the United States over the course of 18 months, between 2017–2019. Photographs reproduced in figures were taken by the author during these visits. This was not an empirical study of campus gardens, but simply an effort to understand and be inspired by the range of food growing opportunities devised by fellow academics. The descriptions are presented to readers on that basis: as possible templates for their own universities, and as persuasive exemplars for university administrators. Visits were made to the Centre for Agriculture and Food Systems at Vermont Law School, the University of Washington Farm, Pomona College Organic Farm, the University of California at Santa Barbara, Los Angeles and Irvine, Chapman College (Orange County), and California State University at Northridge and Dominquez Hills. Site visits were supplemented by investigation of publicly accessible information on university and food garden websites, as well as university governance and policy documents.

## Part II: United States campus food gardens

United States universities are arguably at the forefront of campus food gardens. This was initially a result of the tradition of land grant universities, set up in the nineteenth century to teach and research agriculture, and later to disseminate research to farmers and consumers, (Committee on the Future of Colleges of Agriculture in the Land Grant University System, 1995). Engaging students in practical food growing was a central remit of these institutions. However, today, many United States universities, including those in urban areas, have food gardens as part of the residential liberal arts educational experience. Students can work in food gardens for recreation, course credit or fee remission. Gardens are typically part of the university’s sustainability initiatives and/or curricula, and increasingly they aim to alleviate food insecurity in the student body or adjacent communities (Aftandilian & Dart, 2013)**.**

United States colleges are typically blessed with larger campuses than those in other countries, and offer an immersive, residential experience for students studying general liberal arts programs. As a result, colleges have both the space and the educational culture to foster food growing in curricula. Californian universities have particularly extensive gardens and programs, as a result of the state’s climate, and food composting schemes which feed back into gardens. Some food composting schemes, like that at Pomona College (n.d.-a), were initiated by students decades ago, while others are the result of 2014 amendments to the California *Integrated Waste Management Act of 1989*, which mandated organic waste recycling for businesses and public entities.

### University of California at Santa Barbara

**Fig. 1:**
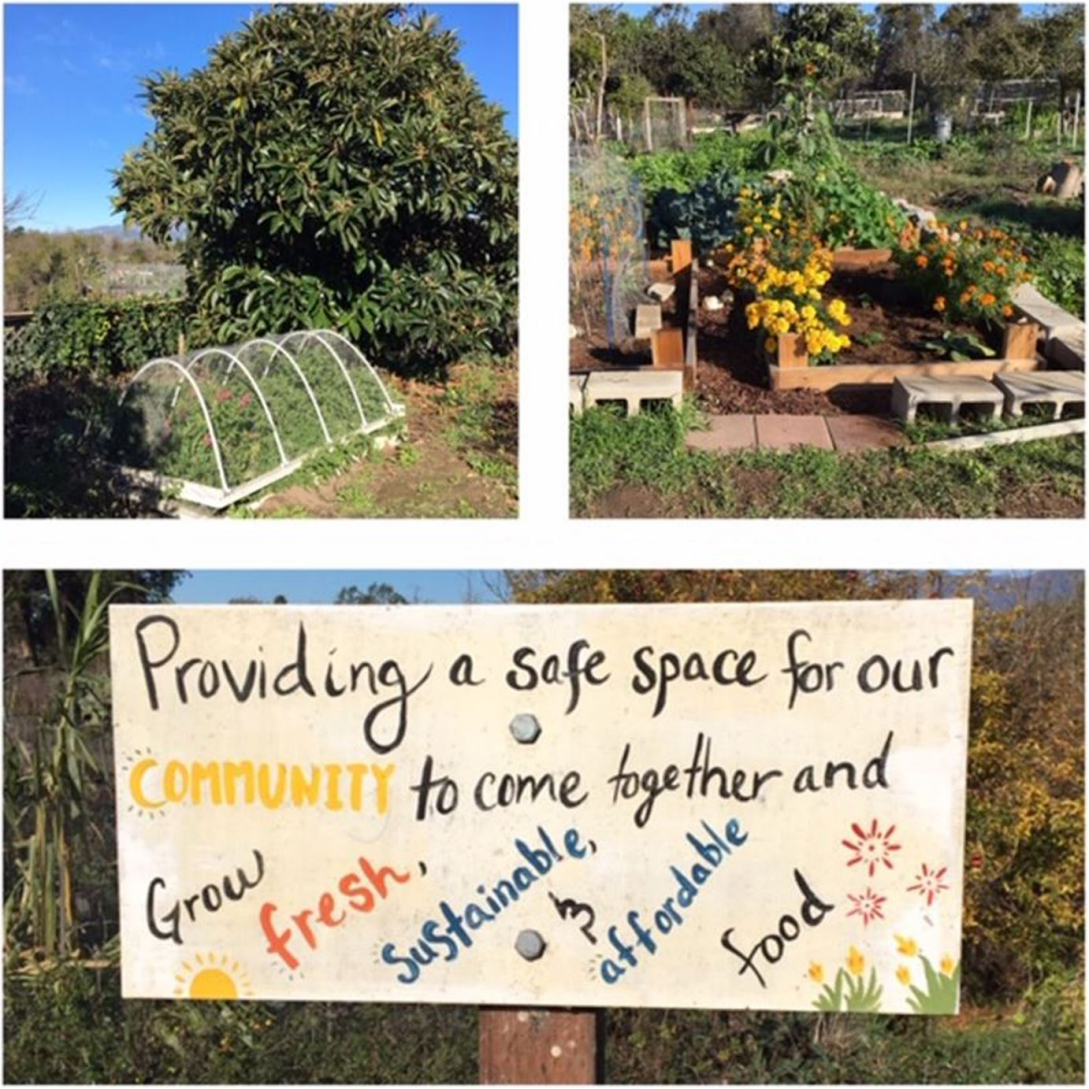
On-site Campus Food Garden, UCSB

The University of California at Santa Barbara (University of California Santa Barbara, n.d.-a) has 14 food gardens on and off campus that repurpose underused space for food production to address food insecurity and to augment environmental education programs across disciplines. Plots are available in an on-site campus garden for staff and students’ personal use, but also for teaching courses as well as individual student projects. Off-campus gardens are attached to student housing, local churches or the local municipality. UCSB has also constructed a campus farm where students can work with local community groups and academics (University of California Santa Barbara, n.d.-b). UCSB offers its students an impressive array of internships, many of which are food and/or sustainability related. The overall sustainability program at UCSB communicates to students and the wider community that the University takes sustainability seriously, and that it is responding appropriately through its own practices, as well as the curricula it offers (see Fig. 1).

### California State University at Northridge

**Fig. 2:**
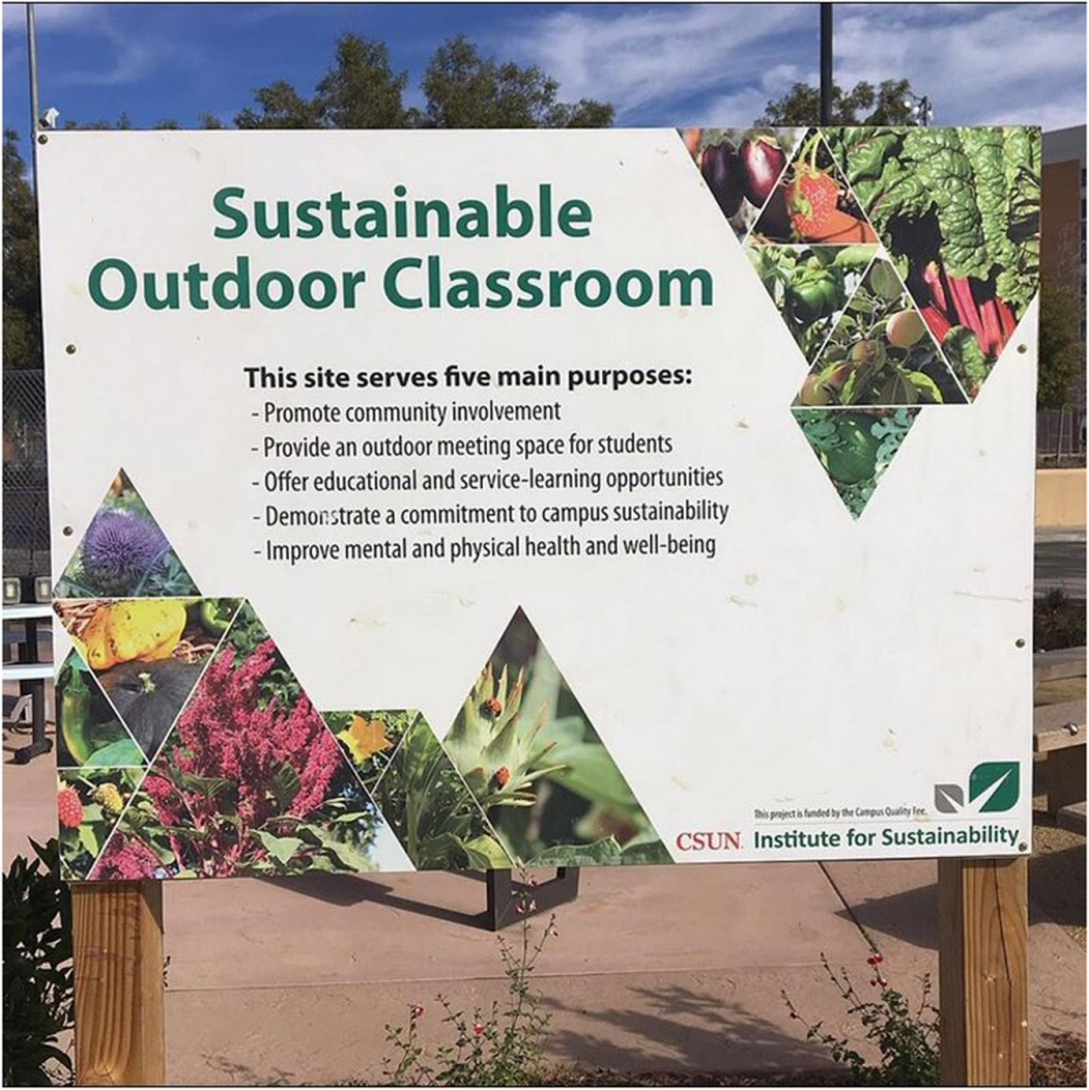
Sustainable Outdoor Classroom Adjacent to Food Garden and Composting site, CSUN

Sustainability is a “key priority” for California State University at Northridge (California State University at Northridge, n.d.-a) that feeds into operations, infrastructure, teaching and research. The University has an Institute for Sustainability, which drives sustainability principles in the curriculum and initiated the University’s 10 year Sustainability Plan. Key achievements of the Institute are an organic food garden which aims to educate “students and the community about sustainable food gardening techniques and healthy food choices, and to promote direct community involvement and service-learning opportunities to students” (California State University at Northridge Institute for Sustainability, 2013, p. 4). The Sustainable Education Garden Centre includes raised beds, a herb spiral, multiple fruit trees, and uses compost from the campus composting scheme (California State University at Northridge, n.d.-b). This processes an impressive 4500 pounds of pre-consumed waste a year, and is run by students who collect food waste from dining halls and process it for service credit. Next to the food garden is the CSUN Outdoor Classroom (see Fig. 2), in which desk power points are connect to adjacent solar panels. Hundreds of students come through the garden each year, learning skills that can be scaled up in a range of situations on graduation.

### University of Washington in Seattle

The University of Washington (UW) in Seattle (University of Washington, n.d.-a) has its own 1.5 acre campus farm, whose mission is to be “the campus center for the practice and study of urban agriculture and sustainability, and an educational, community-oriented resource for people who want to learn about building productive and sustainable urban landscapes.” Students and community members can volunteer at the farm on a weekly basis and students can incorporate farm work into their academic program. (University of Washington, n.d.-b). The farm is available for research projects conducted by students. The farm employs a full-time manager and offers a number of paid and unpaid student internships.

As testament to the farm’s productivity, the farm provides produce to campus housing, food outlets and food banks, as well as a CSA (University of Washington, n.d.-c). CSA stands for “community support agriculture”, a widespread practice in the US. Consumers buy a “share” from a farm at the beginning of a season, which entitles them to produce boxes, dependent on farm availability. If crops fail, consumers do not receive a refund, allowing consumers to share the risks of food growing with farmers.

Perhaps the most impressive part of the UW Farm is the Mercer Court gardens. Mercer Court is a high-rise student housing complex, constructed as five slim line buildings specifically designed to allow southern light to penetrate food gardens (GGN, n.d.). The extensive beds are planted with leafy greens, berries and fruit trees, and demonstrate the food producing capacity of high-density campus and city development if properly planned.

### Pomona College

Finally, Pomona College, one of the top ranked liberal arts colleges in the United States, has an organic food garden with a long and interesting provenance. The farm began in 1998 as a composting program initiated by students who collected dining hall food scraps, mixing them with campus green waste, turning the mix with purpose-built stilts. The farm became the focus for political activity, with students gathering to play music and paint banners before political rallies. University administration seemed to be unaware of the extent of student activity until “they found themselves encountering a rich, vibrant community built around a now almost-farm in [an] abandoned corner of campus” (Pomona College, n.d.-a). Concerned about safety and substance use, the administration tried to shut the farm down, and so began a Save the Farm movement, spearheaded by students, community members and a few “visionary” staff (Pomona College, n.d.-a). Successful lobbying allowed the continuation of the farm, under strict guidelines, and the inception of the first course associated with the land.

**Fig. 3:**
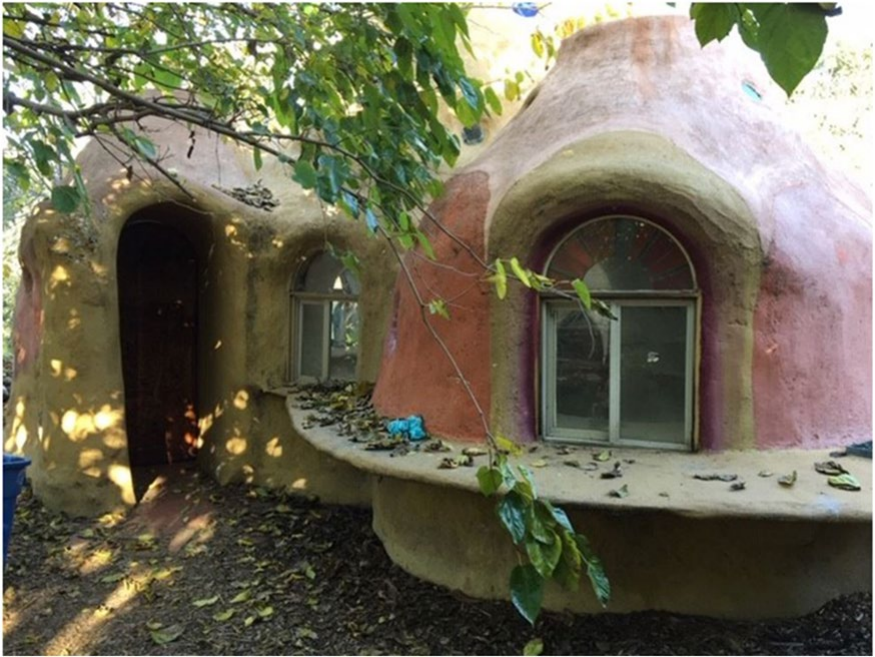
Earth Dome Pomona Organic Farm

The farm now occupies just over an acre of land, with over 200 fruit trees, chickens, vegetable plots, cooking facilities, tables and a stunning, student-constructed earth dome (see Fig. 3). The farm has a full-time farm manager who co-ordinates student employment and volunteers. There are multiple academic courses associated with the farm, as well as independent student projects and theses, (Pomona College, n.d.-b). Visiting in the dying light of a mild California winter’s afternoon, the Farm was a truly magical place.

## Part III: Securing land for the landless – UNSW Campus

Inspired by campus gardens in the United States, a project was initiated to create an urban “farm” on the University of New South Wales (UNSW) Sydney campus under the auspices of the University’s Scientia Education Academy, whose mission is to champion educational innovation and enhance student experience (UNSW Sydney, n.d.-a). Not surprisingly, like other campus gardens, the project faced multiple challenges. Organisation of the campus farm benefitted from prior work on a Faculty garden, developed in the Faculty of Law and Justice courtyards.

### Faculty of Law and Justice Courtyards

**Fig. 4:**
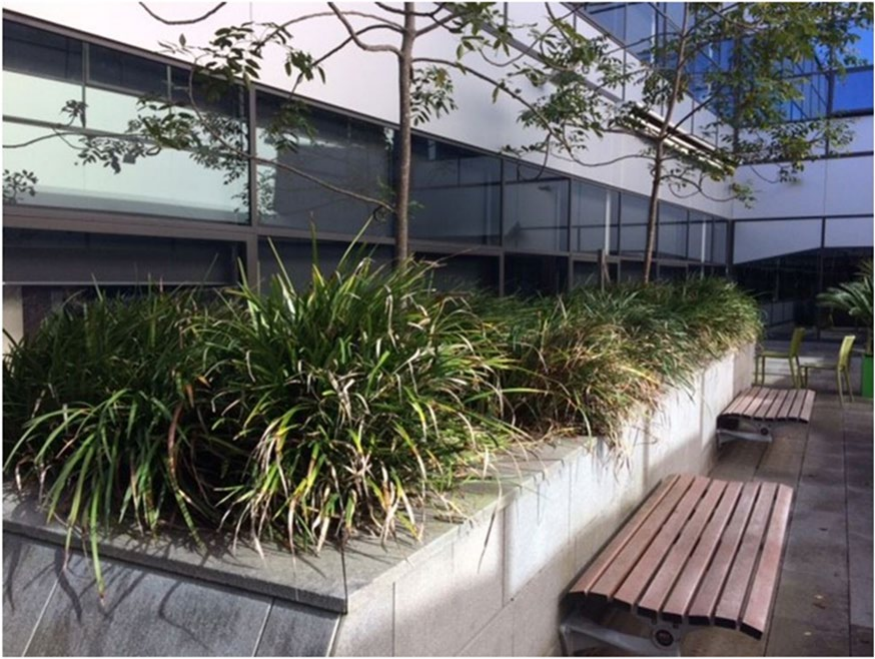
Faculty of Law and Justice Courtyard, UNSW Sydney, Original Dianella Planting, May 2017

The campus farm project had the advantage of being able to showcase to higher levels of university Estate Management, the unit responsible for university buildings and grounds, two existing vegetable gardens in the staff courtyards of the Faculty of Law and Justice. Located on the second floor of the building, these courtyards are technically roof gardens, located above the Law Library. The courtyards include large, raised garden beds that after construction of the building had been planted with *Dianella*, a hardy native flax, ubiquitous in public planting (see Fig. 4). Consisting predominantly of hard, paved surfaces, the courtyards were hot in summer, cold in winter and consequently underused by staff. Faculty leadership was keen for anyone to take an interest in the space. Estate Management was also receptive to staff initiatives, demonstrating existing environmental commitment and knowledge embedded in the University structure. Estate Management removed the compacted *Dianella*, topped the beds with herb and vegetable potting mix and mended the watering system.

**Fig. 5:**
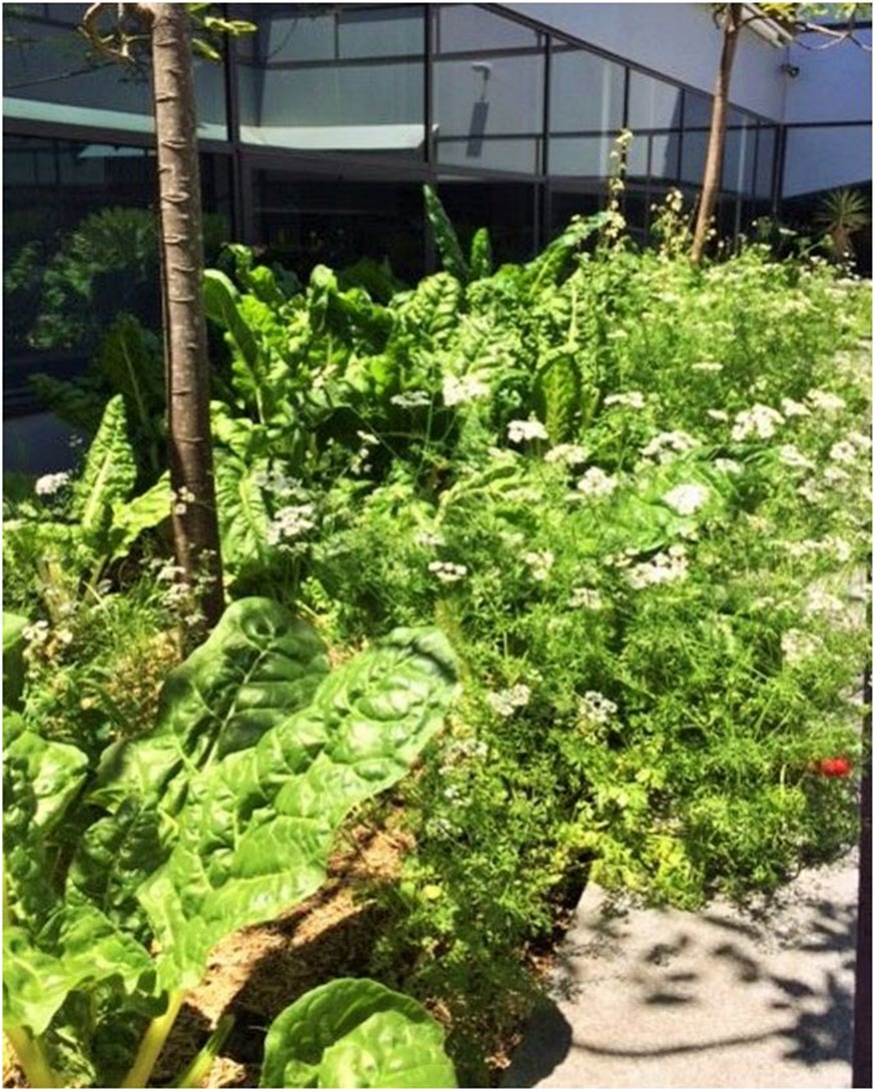
Faculty of Law and Justice Courtyard, UNSW Sydney, Silverbeet, Rocket, Chives, Coriander Flowers, Strawberries, November 2017

The first food garden was established for an elective course taught by the author, People, Land and Community LAWS3115/JURD7515, which covered a range of land-related topics, including green cities, suburban and urban planning, foodways and urban agriculture. Students were invited to volunteer in the garden after class. As a result of building shade and paved surfaces, the garden bed presented challenges common to urban settings (Oke, 2010); the bed received no sun in winter and full sun, as well as intense radiant heat, in summer. Few plants enjoy such conditions. Most vegetables require 6–8 h of sunlight a day, although leafy greens require less. The bed was planted with silverbeet (chard), coriander, mint, rocket, sweet potato and strawberries in late winter 2017. By late spring, it was thriving (see Fig. 5). Staff regularly pick leafy greens for their lunch and dinner, and the garden became a talking point for visitors to the Faculty.

The following year, the Faculty food garden was extended into a southern courtyard, that received more year-round sun. Annual funding, granted to each member of the Scientia Education Academy, was used, not as it might traditionally have been, for a research assistant or the development of online material, but to buy raised beds, plant supports and seedlings. Estate Management again generously donated herb and vegetable growing mix and paid for a watering system to be installed.

Rather than asking students to volunteer, a whole class was set aside for planting (3 h of a 36 h course), scheduled from 1:00 p.m. to 4:00 p.m. on the last Friday of semester. For most students, it was the very last class of their five to seven year combined law degree. Indicative of student engagement, every single student in a class of 45 came and participated with enthusiasm. As they constructed metal raised beds, filled them with soil and planted, they were taught about the University’s bore water supply and the problem of aquifers contaminated from chemical industry south of the University (New South Wales Government, 2018); the importance of clean soil and the advantage Australia has in a global food market as a supplier of food with safe soil provenance (McCarthy et al., 2015); the mistake Sydney is making constructing new housing on our limited, fertile, alluvial soil in our outer suburbs (James, 2014; Malcolm and Fahd 2009); companion planting, worm farming, nitrogen fixing plants and the environmental danger of industrial agriculture’s reliance on chemical fertilisers. Students who had written research essays on urban agriculture contributed their knowledge to the discussion, along with students who had some experience gardening and even foraging, typically with their (often migrant) grandparents. Students were encouraged to note the lack of professional guidance in the construction and planting of the gardens, and to understand that growing food and greening urban environments are activities in which all citizens can engage. The class was served spanakopita from the silverbeet planted by the previous year’s class so current students could enjoy the fruits of urban agricultural labour. For many students it was their first experience eating food that had not originated in a supermarket.

By planting predominantly annual vegetables, the Faculty gardens can be replanted each year by new cohorts of students, (students, like vegetables, are seasonal). Vegetable planting now also forms an important section of the Faculty’s new Food Law LAWS3216/JURD7716 course, which the author co-teaches and convenes.

### UNSW Teaching and Research Garden

Using the Faculty of Law and Justice courtyards as an exemplar, a project was begun to create a UNSW campus “farm.” UNSW is a large research and teaching institution with a full range of disciplines, almost 60 000 students and 7000 academic staff, presenting extraordinary bounty in terms of potential teaching and research projects related to food. After expressions of interest were sought by an email sent to heads of school, a cross faculty working group was formed, ultimately named UNSW Urban Growers (UNSW Sydney, n.d.-b). This was comprised of water engineers in WaterGUM (Water Green-Urban Management); nutrition and chronic disease researchers in Population Health and the George Institute for Global Health; staff in Biological, Earth and Environmental Sciences; staff in Art and Design who had already instigated their own food garden, as well as a textile garden; staff in Environment and Society, Law and Justice, and the School of the Built Environment. Non-academic staff from Sustainability in Estate Management and UNSW Well Being also volunteered their time and resources, along with students from an existing student food growing initiative, The Producers. The project was aided by the UNSW Grand Challenge on Rapid Urbanisation, which provided funding for garden infrastructure and a website, as well as logistical support (UNSW Sydney, n.d.-c).

The first and most obvious task for the group was to secure land. UNSW’s main campus is a 38 hectare site, in a high density, high value neighbourhood, close to Sydney CBD, with limited opportunity for physical expansion. From the outset, it was accepted that the campus farm would constitute multiple small, dispersed sites on the main and secondary campuses, linked by physical signs with a common logo, as well as a website with an interactive map*.* Linked spaces would create opportunities for interdisciplinary research and teaching, as well as showcasing the depth and breadth of food-related work in the University.

The right to use land had to be granted by UNSW Estate Management, which expressed legitimate concern about maintenance of space, and whether it would ultimately bear responsibility for garden beds. Fortunately, some Estate Management staff had already demonstrated a willingness to facilitate food growing, contributing time, skills and resources, and the Faculty of Law and Justice gardens provided living proof to senior levels of Estate Management that staff and students with ongoing academic motivation could maintain space to a high standard.

**Fig. 6:**
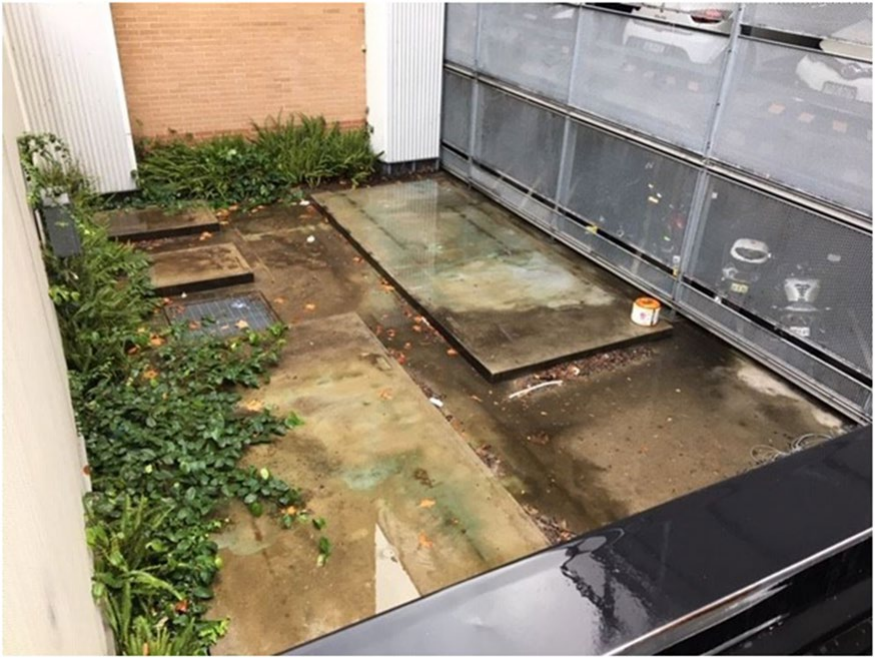
Proposed site of UNSW Teaching and Research Garden, Barker St Carpark, UNSW Sydney, July 2019

Estate Management offered the working group a number of small sites, most of which were not viable, predominantly because of building shade. The working group identified a number of alternative sites, but all were slated for future development. However, one site had great potential, in part because it was so unappealing. It was a north facing slab of concrete, six metres by three metres, inside a metal cage, adjacent to a carpark (see Fig. 6). While the former use of the cage is not known, the presence of self-sown ‘weeds’ indicated the space could support plant life. The secluded nature of the site was potentially advantageous for a working food garden, which unlike the public planting on the rest of the campus, could not look pristine year-round. The working group was mindful that if it could take a genuinely unpleasant urban space and transform it into productive green space, it would demonstrate the potential of urban agriculture to make high density campuses and cities more liveable.

The site was cleared and funding from the UNSW Grand Challenge on Rapid Urbanisation (UNSW Sydney, n.d.-c) and the Scientia Education Academy was used to purchase seven large, raised garden beds that were constructed by students in the Faculty of Law and Justice elective course Food Law LAWS3216/JURD7716. Landscape architecture academics from the School of the Built Environment drew up a design, and UNSW Urban Growers filled and planted the beds with annual and perennial vegetables and herbs. A small trench – the only access to soil beneath the concrete slab – was planted with citrus. The site was named the UNSW Teaching and Research Garden, with the intention that gardens beds would be available on a trimester or yearly basis to academics who wanted to incorporate food growing in their courses or research. Highlighting the importance of environmental education, the garden launch was postponed twice as a result of the 2019–20 Australian bushfires; the air quality in Sydney was so hazardous that the New South Wales Department of Health issued warnings against any outdoor activities.

## Part IV: Lessons learned from the dirt

**Fig. 7:**
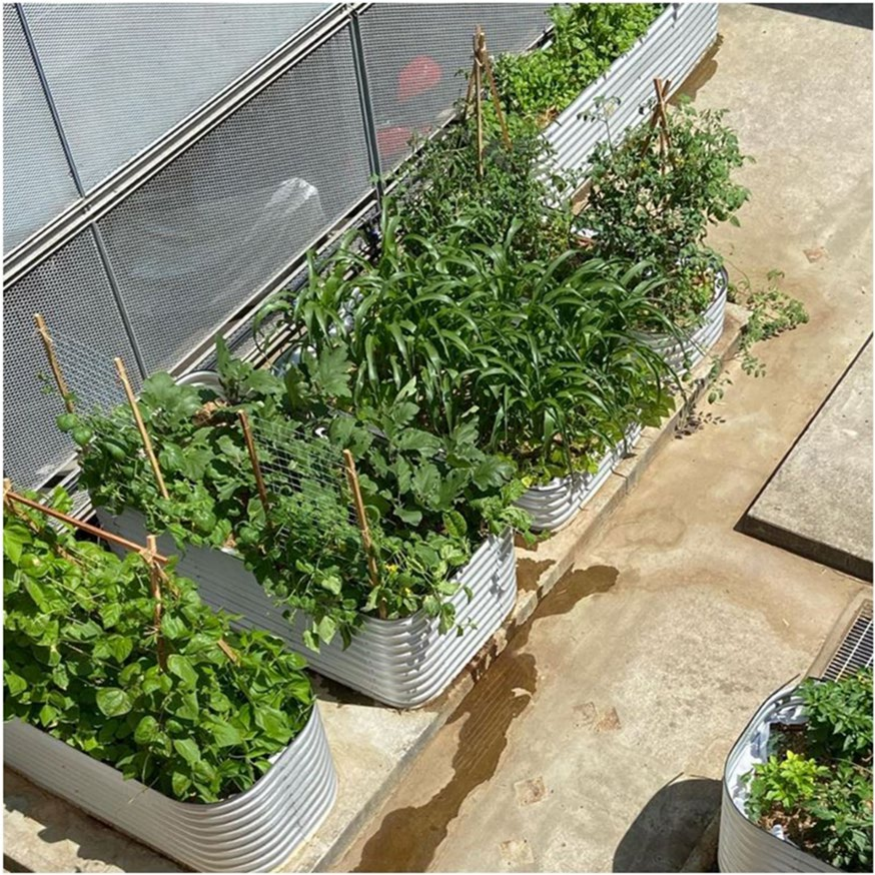
UNSW Teaching and Research Garden, Barker St Carpark, UNSW Sydney, December 2019

The garden flourished over the 2019–20 summer holidays (see Fig. 7) but in early 2020, a global pandemic was declared, and Australian universities shut their campuses. As a result, the UNSW Teaching and Research Garden could not be used for either teaching or research for most of 2020, and again in 2021 as a result of the Delta variant outbreak of COVID-19 and subsequent Sydney lockdown. However, the silver lining of the pandemic was that it allowed time for serious reflection about our food systems, their damaging relationship with nature (Springmann et al., 2018), the vulnerability of our supply chains, and the importance of liveable, sustainable cities. It also highlighted the potential importance of tertiary environmental education through food gardens.

Although there has not yet been an opportunity to conduct an empirical study on the educational impact of the UNSW food gardens, anecdotally students and staff have indicated that planting or simply seeing food growing has increased their knowledge. In the course Food Law LAWS3216/JURD7716, student comments included:

“I enjoyed how practical the components of the course were, especially the gardening and lunch elements. These really helped me feel a part of the class – this is the only class where I've gotten to know the students around me and where I've really felt like I was part of a learning community.”

“Food garden planting [I think this was a great part of the course. It helped split the day up from classroom learning to hands on learning. It was also interesting to learn about gardening and all the processes and techniques required to grow food and helped us understand the reason why we need to understand where food comes from].”

Like most urban citizens, many students have not grown food, and outside of a minority of courses, their formal education is devoid of horticultural content. Through no fault of their own, students are often unaware of the most basic facts about food production; for example, that plants can be reproduced sexually or vegetatively; that the productivity of a plant depends on the soil in which it is grown; that one of the most significant traits of produce in shops is not taste, but transportability; and that all non-animal food is a distinct, unchangeable part of a plant’s life cycle – leaves (spinach, lettuce), followed by flower heads (broccoli, cauliflower), followed by young fruit (beans, cucumbers), mature fruit (tomatoes, eggplant), followed by hard shelled fruit (melons, pumpkins), and finally roots (carrots, potatoes) and seeds (nuts) (Kingsolver, 2007, pp. 64–65). It is arguable that students cannot think critically about seed patents, genetically modified food, global and local food supply and the environmental effects of industrial agriculture, topics common to a range of disciplines, if they do not understand, through some first-hand experience, how food is produced.

Echoing Orr’s argument about environmental education set out at the beginning of this article, interaction with plants, particularly food plants, serves “as a reality check on human possibilities in nature that urban societies presently lack” (Orr, 1994, pp. 117–118). Growing food is hard. It takes time, it is unpredictable, and its success ultimately depends on nature, which we cannot control. As any gardener will attest, our failures are as instructive, if not more instructive, than our successes. The bushfires and pandemic have terrifyingly demonstrated the damage we have done to nature, as well as the uncertainty of our ability to fix this damage. As Orr argued, it is imperative that *all* faculties, not select faculties, make environmental literacy a core graduate skill. The political architects of tomorrow’s climate policies are much more likely to be graduates of law and commerce, than biological or environmental sciences. Environmental literacy cannot come from reading alone; students must interact with nature. Plant blindness has been identified as an impediment to the achievement of United Nations’ Sustainable Development Goals, which most universities have committed to, including UNSW. In this regard, it has been argued that “Universities are by definition a context in which innovatory and unconventional educational approaches can be established in order to construct knowledge and change attitudes” (Amprazis & Papadopoulou, 2020, p. 1078). University food gardens are one such innovation, demonstrating genuine commitment to change beyond paper statements.

Most of our students will never become farmers, but they will become residents and workers of urban centres that must be liveable and sustainable, particularly if we face the prospect of future public health lockdowns and restrictions on movement. Human beings’ evolutionary preference for natural environments (Wilson, 1984) means that liveable cities are green cities. Extensive research demonstrates the role that green space plays in physical and mental health, (Gamble et al., 2014; Gascon et al., 2016; Kaplan & Kaplan, 1989; Kaplan, 1995; Pereira et al., 2012; Ulrich et al. 1991), neighbourhood satisfaction (Arnberger and Eder 2012; Douglas et al., 2019), and even reduction of crime. (Fleming et al., 2016; Kuo and Sullivan 2001) Green space helps to combat urban heat islands (Bowler et al., 2010; Hunter et al., 2014; Rayner et al., 2010), reducing greenhouse gasses from artificial cooling, as well as excess deaths from city heat waves (Kovats & Elbi, 2006). While public parks and street plantings play a significant role in urban greening, most space in Australian cities is private, not public: freestanding housing lots, terraces, residential and commercial strata schemes. The owners and residents of that private property need skills to green their own space, so they are not exclusively reliant on councils, developers and professional landscape architects to do so. As a consequence of building shade, hard surfaces, water run-off and radiant heat, greening urban space is not easy; like us, plants did not evolve surrounded by concrete and glass. By engaging students in university food gardens, particularly on high-density campuses that are essentially cities in miniature, we are arming students, including those outside the physical and biological sciences, with the knowledge and skills to overcome urban greening challenges. If they can participate in the production of food from a concrete cage or a high-rise roof, they will know what is possible in their future workplaces and homes.

University food gardens build community and generate joy. Although the express aim of the UNSW Teaching and Research Garden was to create individual garden beds for individual academics to utilise in teaching and research, the project has steadfastly remained a communal endeavour of students, academics and professional staff who are committed to collective growing and environmental sustainability, particularly through the pandemic lockdowns. Support has been given from kindred green spirits at the highest levels of the University through to the private company contracted to maintain university grounds. The University food gardens also give pleasure to those who simply see them. Exclamations of delight can be heard in the Faculty of Law and Justice courtyards when people identify spinach or mint, and staff share pictures of meals they have cooked from produce gathered at the end of a stressful day. After 2020–2021, the value of community and joy should not be underestimated by any educational institution.

## Conclusion

2022 will hopefully see a revitalisation of the UNSW food gardens. Plans had been made to house native bees inside the Teaching and Research Garden, and chickens have been mooted. These will be further explored, along with potential student projects for food recycling and worm farms, planting of donated wicking beds and bi-monthly working bees.

Beyond the work conducted at UNSW, university food gardens will continue to face challenges, including a lack of space and an academic culture that insufficiently values “doing” that goes beyond reading and writing. The COVID-19 pandemic has presented new challenges, in particular the rise of online learning, with the tempting potential for universities to earn fees from thousands of students who could never physically attend campus, even once the pandemic is over. However, the pandemic has also highlighted the value of urban food gardens to supplement disrupted supply chains, as spaces for outdoor recreation, and to create those all-important social bonds that help communities and institutions survive crises. Campus gardens can perform all these roles, in addition to ensuring that our students graduate with genuine levels of environmental literacy. The creation of campus gardens for teaching and research does not require professional expertise, nor need gardens be limited to courses in biological sciences. By following the example of established campus gardens, particularly in the United States, academics from a range of disciplines can instigate successful food garden projects.

## Supplementary Information

Below is the link to the electronic supplementary material.

Supplementary file1 (DOCX 27 KB)

## Data Availability

Not applicable.

## Footnotes

1 In her record of her family’s year living only on home grown and locally sourced food, Barbara Kingsolver recounts a story of a young boy who used to watch her husband growing food in the yard of his city home. As her husband pulled carrots from the ground, the boy exclaimed, “How’d you get them *in* there?” (Kingsolver, 2007, p. 12).
