# Supplementary material for: Learning from the Dirt: Initiating university food gardens as a cross-disciplinary tertiary teaching tool
Source: Journal of Outdoor and Environmental Education. 2022 May 13;25(2):199–217. doi: 10.1007/s42322-022-00100-6 (PMC9098788; doi:10.1007/s42322-022-00100-6)
Supplement: Supplementary file 1 — Supplementary file1 (DOCX 27 KB) [file 42322_2022_100_MOESM1_ESM.docx]

Response to reviewers ‘Learning from the Dirt: Initiating university food gardens as a cross-disciplinary teaching tool

| Reviewer 1 |  |  | |
| --- | --- | --- | --- |
| 1. needs greater exploration of the literature around experiential and outdoor learning in Australian universities, particularly in the physical and biological sciences | Clarified | The article is not about experiential and outdoor learning in Australian universities and it is not about the physical and biological sciences. The statements about experiential and outdoor education are made specifically with reference to ‘arts, law and social sciences’. The first paragraph of Part I has been amended to make it clearer that the Part is about university education across all disciplines, and the comments are made with reference to the entire project of higher education. Also, ‘outdoor learning’ has been changed to learning outside to remove any confusion with the discipline of outdoor education.  The article is not an empirical study and so the template for empirical research has not been used. In empirical research, a literature review demonstrates an author’s knowledge of previous research findings, and possible gaps in the literature. In non-empirical research, it is not the norm to reference all or most previous work in the area, but rather to cite work as and when it is relevant. For example, a work like Orr’s is not based on empirical research at all. The book is a collection of articles that make normative arguments in favour of environmental education. | |
| Some of the claims around the lack of practical components in courses simply don't stack up against the reality of more vocationally-aligned degree programs. Especially as there are examples of campus-food gardens in Universities that are directly linked to curriculum. | Rejected | This part of the article is making a high-level argument about university courses generally. Of course, there are exceptions to the general rule in vocationally-aligned degrees, but that does not invalidate the fact that the vast majority of university teaching, particularly in law, arts and social sciences, occurs in classrooms. Most university degrees are not vocationally aligned, and of those that are, many are still not taught practically or outside eg law and medicine. Further, courses that traditionally had practical and/or outdoor components, have had these reduced in recent decades eg engineering, landscape architecture and architecture.  While the author has tried to find examples of universities in Australia that have academic courses specifically tied to university food gardens (as opposed to community gardens on campus), none have been found. While the reviewer states that this is the case, no examples were given. There are of course examples from overseas which have been referenced. | |
| The background/scene setting around the lack of knowledge around food origin and growing of students entering universities is not exactly the right question here. More critical is the how students increasingly have little understanding of 'nature' - the comments on 'plant blindness' in the paper bear this out. These are complex issues and deserve to be discussed in greater detail (i.e. student learning and connections to nature) to provide a better context for the university food garden. | Clarified. | The argument has been clarified at p 3. The argument of this section is not about nature generally (which other writers have addressed well eg AO Wilson) or plant blindness. It is about food growing, which has a particular significance for ecological literacy. Hopefully that significance has been clarified.  Teaching in the area of food law and policy, and urban agriculture, lack of knowledge of food growing is a central problem. That is connected to a lack of knowledge of nature, but it is more specific. Understanding how a forest grows does not necessarily help students understand how food traditionally reached people’s tables and how it reaches people’s tables today. | |
| what are the key attributes or elements that have been successful for these gardens in USA Universities and what are the drivers for success? This could then position the 'results' of the project outlined later for the UNSW garden | Clarified | The author is not in a position to judge what elements have been successful or what the drivers for success are in the US campus gardens because no research was done in this regard. The US gardens were simply used as exemplars that demonstrate that universities can contain food gardens. While this is not as ambitious as the reviewer’s suggestion, the very idea of a food garden on a university campus in Australia is still unusual. Being able to point to the existence of gardens in the US (where they are commonplace) is of benefit to universities like UNSW that had no food gardens. Clarification added at p3. | |
| I'm guessing these would have been required in the grant application or request for funding from the University's Scientia Education Academy? | Partially accepted | Funding clarified – there was no request for funding. It is ‘as of right’ funding given to all members of the Academy to spend on their own projects.  The Teaching and Learning Garden was also funded by the Scientia Education Academy and the Grand Challenge on Rapid Urbanisation which is already stated on p.13 and 14 | |
| Was there any technical support or expertise sought in preparation of the courtyard garden? If so how was this facilitated? Some of the images do not inspire great horticultural expertise, which is fine as its not the focus here, but how the garden drew from knowledge and skills needs explanation. | Clarified | There was no professional expertise involved in the construction and planting of the Law School courtyard gardens, other than the author’s 30 years of home gardening. Clarification added p 12-13. The article also makes it clear at p18 that lack of professional training is an important part of university gardens; that is, food gardens can be created by staff and students in all disciplines, not only in disciplines with horticultural expertise.  The article already specifies at p 14 .that the Teaching and Learning Garden bed design was done by landscape architecture academics from the School of the Built Environment. | |
| o An introduction to the University and the estate. This is provided later (p.14) but is needed here to provide a context to the garden. This would be aided by some form of map and perhaps the use of less (more directed and relevant) images | Partially accepted. | - There is a description of the university provided at p13 already. - The role of Estate Management has been clarified at p9 - A map seems unnecessary as both the Law School and Barker St Car Park can be located on a map of UNSW if readers are interested, but their location relative to other buildings on campus is of limited relevance, particularly in the case of the Law School. - It is not clear what ‘less (more directed and relevant) images’ means. | |
| How was this group formed? It would be useful to know how the garden activities themselves became part of curricula (i.e. "Vegetable planting now also forms an important section of the Faculty's new Food Law course"). Statements such as this need explanation and evidence. | Accepted | Clarification included.   - The group was formed after an email was sent to HOS asking for expressions of interest. - The author convenes and teaches the courses in which the gardens are currently used. The pandemic has prevented their use in other courses as yet. | |
| What have been the main drivers in initiating the project, sustaining it, who are the champions across disciplines and what is the future of the garden? | Rejected | This is already clearly stated in the article on p9: *“*Inspired by campus gardens in the United States, **the author initiated a project to create an urban ‘farm’ on UNSW campus** under the auspices of the University’s Scientia Education Academy, whose mission is to champion educational innovation and enhance student experience, (UNSW, n.d.).” and at p 13  “A cross faculty working group was formed, ultimately named UNSW Urban Growers (Sustainability UNSW Sydney, n.d.), comprising water engineers in WaterGUM (Water Green-Urban Management) etc”.  The garden has been sustained by a group of students, academics and non-academic staff: stated at p18.  The future of the garden is clearly stated in the conclusion, which has now been updated to take into account the lack of access to campus in 2021 because Sydney has been in lockdown for a substantial period. Access to campus has been barred for the best part of 2 years. | |
| Some useful details provided on student engagement and anecdotes but there is a distinct lack of evidence about how the garden has provided environmental education outcomes, either formally, through curricula, or informally through engagement and activities? A key question here is what have been the main education outcomes from the garden and what is the evidence to support these conclusions? | Partially accepted | The title of the article has been changed to “Learning from the Dirt: Initiating university food gardens as a cross-disciplinary tertiary teaching tool” rather than ‘using university food gardens….” ‘Initiating’ is a more accurate term for what the article aims to do.  There is limited evidence of educational outcomes yet. The gardens are new. They were started in 2018-19 and the pandemic has prevented their use in 2020-21 because of Sydney’s lockdowns and universities all teaching online. This is made clear at p17.  The article clearly states that it is not an empirical study. At the beginning it states:  “The article is not an empirical study of the effectiveness of a particular garden(s), rather it is a descriptive and persuasive piece that aims to inspire academics, from all disciplines, particularly those outside biological sciences, to instigate food gardens on their own campuses, in order to increase graduate environmental literacy” and at the end it notes the article’s limitations in terms of empirical findings:  “First, although there has not yet been an opportunity to conduct an empirical study on the educational impact of the UNSW food gardens, anecdotally students and staff have indicated that planting or simply seeing food growing has increased their knowledge.” P17 | |
| Essentially this is a case study paper, exploring the development and use of a campus food garden, I'd suggest re-writing the paper so it reflects a case study method and structure in any future revision. | Rejected | The article was very clear that this is not a case study. It is not empirical research that was designed to demonstrate educational outcomes. It would be unethical to retrofit or rewrite the article to make it look as though it has empirical ‘findings’ because it does not.  The Journal’s description includes “The journal provides an academic forum in which empirical and non-empirical research concerning outdoor and environmental education can be shared and discussed.” The author accepted the Journal’s invitation to submit a non-empirical piece and as a result, the article does not have empirical findings. | |
| There is also very limited use of the relevant literature, particularly studies exploring University or Campus food gardens. Examples include Duram and Klein (2015); Eugenio-Gozalbo et al (2020); and Marsh et al 2020). The latter is an Australian study | Partially accepted. | - Duram and Klein (2015) is referenced at the beginning of the article, along with a number of other studies of campus gardens but Duram was accidentally omitted from the reference list. Corrected. - Neither of the 2020 studies mentioned by the reviewer were published when this article was written, although they have been included now at the beginning of the article.   However, as stated above, in non-empirical research, it is not the norm to reference all or most previous work in the area, but rather to cite work as and when it is relevant. For example, while excellent, the Marsh article is about a community garden on a campus. This article is not about community gardens, it is about teaching gardens, and so an article about community gardens is not strictly relevant. Nor are a whole range of other articles about campus gardens, with which the author is familiar, but as they do not bear directly on this article’s arguments, they have not been cited. That is the academic norm in non-empirical research. | |
| This section needs to frame the 'results' in a more meaningful discussion around 'university food gardens' per se, rather than the broad brush of green space and green infrastructure benefits that are outlined on page 19. | Rejected | As above. This is not an empirical article and normative arguments make up a core component in many academic disciplines. | |
| **Reviewer 3** |  | |  |
| Perhaps, a definition of food garden/ campus garden would be needed, since there were different types of examples (gardens) described in the manuscript. | Accepted | | Definition included in opening paragraph |
| Generally, the manuscript was loaded with quite a lot of personal experience and judgemental upbringings. Perhaps, the author may consider use a phenomenological rhetoric, a first-person presentation to depict the message how green garden can be a teaching learning resource in the premise of the data (portrayed here) in this manuscript. | Rejected | | The journal descriptions includes: “The journal provides an academic forum in which empirical and non-empirical research concerning outdoor and environmental education can be shared and discussed.”  There is a huge range of academically-acceptable ways of writing across disciplines. In the author’s discipline, some personal observations are acceptable and normative arguments (‘judgemental upbringings?’) are the norm. |
| For the part II, it appears a travel monologue. It could be acceptable in current format, but a more systematic and critical review on how these experience could be translated into the lesson learnt should be presented in the Part IV. For example, each venue in various universities will communicate different message to anyone who engages there. Do you see the differences, and what about your comparisons here? You may wish to address this conflict, if you find in the paper! | Clarified | | The US gardens are simply presented as an exemplar. They are included to alert readers who may be unfamiliar with university gardens to the widespread existence of gardens. The advantage of descriptions of real gardens and links to university websites, is that unlike (for example), references to research on gardens, readers and university administrators to whom they report, can see examples of functioning gardens. Clarification added at p3. |
| The manuscript focused more on the physical intrastructures of the campus garden. While the manuscript was framed under environmental education, perhaps there should be more discussion on what way (e.g. pedagogy) these hardware were used to nurture student's environmental literacy, behaviors or even the action skills. For examples, what kind of experiential and outdoor learning activities involve in farm to influence students' ecological literacy? How about the competency of the teachers used the campus garden for teaching? | Accepted | | The title of the article has been changed to “Learning from the Dirt: Initiating university food gardens as a cross-disciplinary tertiary teaching tool” rather than ‘using university food gardens….” ‘Initiating’ is a more accurate term for what the article aims to do.  There is limited evidence of the pedagogical impact as yet. The gardens are new. They were started in 2018-19 and the pandemic has prevented their use in 2020-21 as a result of Sydney’s lockdowns.  They are currently used for two courses that the author teaches, and while there were plans to use the gardens in other courses, the pandemic shutdowns have prevented this. |
